# Evaluation of neutrophil gelatinase-associated lipocalin in pediatric patients with acute rotavirus gastroenteritis and dehydration

**DOI:** 10.1186/1824-7288-39-52

**Published:** 2013-09-03

**Authors:** Tanju Çelik, Emel Altekin, Rana İşgüder, Yasin Kenesari, Murat Duman, Nur Arslan

**Affiliations:** 1 Department of Pediatric Emergency, Dr. Behcet Uz Children Hospital, Izmir, Turkey; 2 Dr. Behcet Uz Children Hospital, Dokuz Eylul University Faculty of Medicine, Izmir, Turkey; 3 Division of Pediatric Emergency, Dokuz Eylul University Faculty of Medicine, Izmir, Turkey; 4 Pediatric Gastroenterology, Hepatology and Nutrition, Dokuz Eylul University Faculty of Medicine, Izmir, Turkey

**Keywords:** Acute kidney injury, Children, Dehydration, Neutrophil gelatinase-asssociated lipocalin

## Abstract

**Background:**

Dehydration caused by acute rotavirus gastroenteritis is a frequent finding in pediatric patients. The most important treatment modality in these patients is recognising and treating dehydration, electrolyte imbalance and acute kidney injury. Neutrophil gelatinase-asssociated lipocalin (NGAL) is used widely as a biomarker for the diagnosis of acute or chronic renal injury in numerous clinical studies. It is recognized as an early marker of acute renal failure before the elevation of routine biochemical tests such as creatinine. The aim of this study is to investigate the plasma and urine NGAL concentrations in mildly or moderately dehydrated patients with acute rotavirus gastroenteritis.

**Material and methods:**

A total of 30 patients (13 girls, mean age 62.5 ± 46.2 months) with diarrhea and mild/moderate dehydration and 35 healthy controls (17 girls, mean age 81.1 ± 41.8 months) were enrolled in the study. Plasma and urine NGAL levels of the two groups were compared.

**Results:**

The mean age, gender and serum creatinine levels of the patients and healthy controls were similar. The mean plasma and urine NGAL levels of the patients were significantly higher than controls (plasma: 118.6 ± 81.2 vs. 66.5 ± 11.3, p = 0.001 and urine: 17.7 ± 17.5 vs. 10.6 ± 7.9, p = 0.035, respectively).

**Conclusion:**

Mildly or moderately dehydrated children have higher plasma and urine NGAL levels compared to control subjects. Plasma and/or urine NGAL levels can be used for the early prediction of renal impairment in children with mild or moderate dehydration.

## Background

Acute dehydration caused by rotavirus gastroenteritis is seen frequently in pediatric age group. The most important treatment modality in these patients is recognizing and treating dehydration, electrolyte imbalance and acute kidney injury.

Although, serum creatinine level is widely used for the diagnosis of acute renal failure, in fact, it is a poor marker for acute renal injury [1,2]. Its generation, distribution and excretion can be regulated by many factors in the body [2]. Since, serum creatinine level is slowly rising after acute renal injury; new biomarkers are needed for the early diagnosis.

Human “Neutrophil gelatinase-asssociated lipocalin” (NGAL, lipocalin 2) was originally identified as a 25-kDa protein covalently bound to gelatinase from neutrophils [3,4]. It is mainly expressed by neutrophils and various epithelial cells, and variable degrees of NGAL gene expression is demonstrated in human tissues like uterus, prostate, salivary glands, lung, trachea, stomach, colon, and kidney [3,5]. It was shown that NGAL was a potent early biomarker of renal injury in numerous clinical studies [6-12].

There is only one adult study in the literature investigating serum NGAL levels in patients with acute gastroenteritis [13]. In this study, serum NGAL levels were shown to be higher in dehydrated patients compared to healthy controls. To the best of our knowledge, there is not a pediatric age group study investigating plasma or urine NGAL levels in dehydrated patients.

The aim of this study was to investigate the plasma and urine NGAL concentrations as an early biomarker of prerenal acute kidney injury in mildly or moderately dehydrated pediatric patients with acute rotavirus gastroenteritis.

## Patients and methods

A total of 30 patients (13 girls and 17 boys, mean age 62.5 ± 46.2 months) with diarrhea and mild/moderate dehydration were enrolled in the study. Complete physical examinations were performed for all of the patients. Mild and moderate dehydration was defined according to American Academy of Pediatrics (AAP) guidelines [14]. According to the AAP, mild dehydration is described as a 3%–5% loss of total body fluids and moderate dehydration represents a 6%–9% loss of total body weight.

Chronic hepatic, intestinal, renal, neurologic, metabolic and immunologic diseases and drug usage for any disease were considered as criteria for exclusion. Stool microscopic examination and culture for bacteria and antigen tests for parasites were performed for every patient and patients with bacterial or parasitic gastroenteritis were also excluded from the study. Thirty five age- and sex-matched healthy children who were admitted to well child outpatient clinic for routine visits constituted the control group (17 girls and 18 boys, mean age 81.1 ± 41.8 months).

Rotavirus was determined in fresh stool samples using the highly sensitive and specific ELISA test (Rota Adeno Antigen Test Device, Cambridge). Blood and urine samples were collected on admission to the hospital before the rehydration therapy. Complete blood count, serum blood urea nitrogen (BUN), creatinine and C-reactive protein levels were investigated for all the patients. 2 ml venous blood were drawn from a peripheral vein in EDTA containing tube and 5 ml urine sample were obtained for measurement of plasma and urine NGAL levels, respectively. Plasma and urine samples were obtained at the same time and stored at -80°C till the day of analysis of NGAL.

NGAL levels of plasma samples were measured using the Triage NGAL assay (Biosite, Inverness Medical Innovations, San Diego, CA, USA). The Triage NGAL test is a point-of-care, fluorescence-based immunoassay for the rapid quantitative measurement of NGAL concentration in EDTA-anticoagulated whole blood or plasma specimens. The precision and sensitivity of the assay have been described [15]. Concentrations of NGAL in urine samples were determined by a quantitative kit on an Architect 16000 analyzer (Abbott Diagnostics). The assay is a two-step (sandwich) assay using Chemiluminescent microparticle immunoassay technology. The ARCHITECT assay had a functional sensitivity 2 ng/mL (20% coefficient of variation [CV], 95% confidence) and total CVs < 5.0% [16].

### Ethical approval

The study protocol was designed in compliance with the Declaration of Helsinki. Informed consent was obtained from both the children and their parents on enrollment in the study. The study was started after the approval of the Ethics Committee.

### Statistical analysis

Statistical analysis was performed using the Statistical Package for Social Sciences (SPSS) version 15.0. Data were expressed as mean ± standard deviation. Kolmogorov-Smirnov test was used to examine normal distribution of the population. Student *t* test and Mann–Whitney U-test were used for comparing group averages. Chi-square test was used for comparing group ratios. Interco relations between parameters were computed through the Pearson’s correlation analysis. Correlation coefficient indicated low correlation at 0.10–0.29, medium correlation at 0.30–0.49, and high correlation at ≥0.50. All *p* values were two-tailed and group differences or correlations with *p* <0.05 were considered to be statistically significant.

## Results

The mean age and gender of the patients and healthy controls were similar (p = 0.137 and p = 0.804, respectively) (Table 1). The mean hemoglobin levels, platelet count and creatinine levels were not different in two groups (Table 1). On the other hand, the mean white blood cell count, BUN and C-reactive protein levels was significantly higher in patients when compared to healthy controls (p = 0.001, p = 0.047 and p = 0.012, respectively) (Table 1).

**Table 1:** Comparison of clinical and laboratory parameters between dehydrated patients and healthy controls (mean ± SD)

**Parameter**	**Dehydrated patients (n = 30)**	**Healthy controls (n = 35)**	***p *value**
**Age (months)**	62.5 ± 46.2	81.1 ± 41.8	0.137
**Gender (F/M)**	13 / 17	17 / 18	0.804
**Hemoglobin (g/L)**	12.2 ± 1.7	12.6 ± 1.1	0.273
**Leukocyte (10**^**3**^**/μL)**	13.7 ± 6.2	8.8 ± 1.7	**0.001**
**Platelet (10**^**3**^**/μL)**	359.0 ± 128.1	316.2 ± 82.2	0.151
**C-reactive protein (mg/L)**	2.6 ± 4.1	0.2 ± 0.1	**0.012**
**Blood urea nitrogen (mg/dL)**	15.8 ± 11.3	11.0 ± 3.2	**0.047**
**Creatinine (mg/dL)**	0.55 ± 0.2	0.53 ± 0.2	0.609
**Urine NGAL (ng/mL)**	17.7 ± 17.5	10.6 ± 7.9	**0.035**
**Plasma NGAL (ng/mL)**	118.6 ± 81.2	66.5 ± 11.3	**0.001**

The mean plasma and urine NGAL levels of the patients were significantly higher in patient group than controls (p = 0.001 and p = 0.035, respectively) (Table 1). When patients were divided into two dehydration groups, significantly high urinary NGAL levels were detected in the moderately dehydrated group compared to mildly dehydrated patients (p = 0.001) (Table 2). Similarly, plasma NGAL levels were also high in the first group but the difference was not statistically significant (p = 0.295) (Table 2).

**Table 2:** Plasma and urine NGAL levels and other laboratory parameters in mildly and moderately dehydrated patients (mean ± SD)

	**Mildly dehydrated (n = 20)**	**Moderately dehydrated (n = 10)**	***p *value**
**Leukocyte (10**^**3**^**/μL)**	12.7 ± 5.5	15.6 ± 7.5	0.375
**C-reactive protein (mg/L)**	1.9 ± 3.1	3.6 ± 5.2	0.235
**Blood urea nitrogen (mg/dL)**	13.1 ± 3.8	17.1 ± 13.5	0.561
**Creatinine (mg/dL)**	0.57 ± 0.18	0.52 ± 0.14	0.668
**Urine NGAL (ng/mL)**	9.2 ± 6.5	34.7 ± 20.2	**0.001**
**Plasma NGAL (ng/mL)**	103.3 ± 57.6	149.2 ± 112.5	0.295

There was a positive correlation between urine and plasma NGAL levels (r = 0.372, p = 0.002) (Table 3). Urine NGAL levels were also positively correlated with leukocyte and C-reactive protein levels (r = 0.324, p = 0.017 and r = 0.350, p = 0.023, respectively). Similarly, plasma NGAL levels were positively correlated with C-reactive protein levels (r = 0.340, p = 0.027) (Table 3).

**Table 3:** Correlation between laboratory parameters in patients and controls (n = 65)

	**U-NGAL**	**P-NGAL**	**Leukocyte**	**C-RP**	**BUN**
**P-NGAL**	.372**				
**Leukocyte**	.324*	.178			
**C-RP**	.350*	.340*	.174		
**BUN**	.124	–.031	.194	.190	
**Creatinine**	–.098	.036	.036	.044	.333*

*Correlation is significant at the 0.05 level.

**Correlation is significant at the 0.01 level.

## Discussion

This study demonstrated that mildly and moderately dehydrated patients with acute gastroenteritis and with normal creatinine levels had significantly higher urine and plasma NGAL concentrations than the healthy controls. Besides, this study revealed that patients with moderate dehydration had significantly higher urinary NGAL concentrations than mildly dehydrated ones. To the best of our knowledge, this is the first study investigating urine and plasma NGAL levels in pediatric patients with acute gastroenteritis.

Results of studies which investigating the serum NGAL levels for diagnosis of acute renal failure or acute kidney injury are inconclusive. In studies including critically ill patients both adult and childhood age groups, blood and urinary NGAL levels did not predict acute renal injury earlier than serum creatinine levels [17-19]. On the other hand, in other studies consisted adult and pediatric patients showed that the patients with acute renal injury had elevated mean urinary or plasma NGAL levels in early stages [7-12].

There is only one study in the literature investigating serum NGAL levels in adult patients with acute gastroenteritis [13]. In this study, Antonopoulos et al. showed that 12 mildly dehydrated patients had significantly higher serum NGAL levels than the healthy controls (129.4 ± 25.7 ng/mL and 60.6 ± 0.4 ng/mL, respectively). Leukocyte count, blood urea nitrogen and serum creatinine levels were not statistically different. They did not investigate urinary NGAL levels in their study. Depending to these results, the authors suggested that serum NGAL levels could be a very sensitive biomarker for prerenal kidney dysfunction even in early stage [13]. Similarly, creatinine levels of patients did not different from the healthy controls in our study. On the other hand, serum BUN levels of our patients were slightly higher than the controls.

In dehydration, similar to other reasons reducing renal blood flow, glomerular filtration rate may decrease and glomerular epithelial cells can be damaged [2,13,20]. In this situation, injury of the brush border of proximal tubule cells is the earliest lesion in the prerenal stage and during this early period minimal acute tubular necrosis cannot be detected using routine laboratory investigations such as serum creatinine levels [2]. Dehydration as a form of prerenal acute kidney injury may be represented by a tubular enzymuria and a concomitant increase in serum NGAL [2,13]. Although NGAL is expressed only at very low levels in several human tissues, it is markedly induced in injured epithelial cells, including the kidney [21]. NGAL is increased in urine very early (2 h) after injury; hence, NGAL may be a sensitive biomarker to detect minimal acute kidney dysfunction as seen in dehydrated patients with gastroenteritis. The higher urinary NGAL concentrations in patients with moderate dehydration than the mildly dehydrated ones in this study considered that the degree of dehydration affected the severity of acute kidney dysfunction.

One major limitation of our study is the small number of patients. We believe that difference in both urinary and plasma NGAL levels could be more prominent in a larger patient group with mild-moderate dehydratation. Another limitation is the cross-sectional, case- control design of the study. A prospective study analyzing the alterations of NGAL levels before and after rehydration therapy is needed. Finally, simultaneous evaluation of a glomerular filtration rate would be beneficial to assess renal functions in these patients.

## Conclusion

This study showed for the first time that mildly or moderately dehydrated children have higher plasma and urine NGAL concentrations compared to control subjects even in early stage with normal creatinine levels. Plasma and/or urine NGAL levels may be used for the early prediction of renal impairment in children with mild or moderate dehydration.

## Competing interest

The authors declare that they have no competing interests.

## Authors’ contributions

NA, TÇ, Rİ have primary responsibility for protocol development, patient screening, enrollment, data analysis and writing the manuscript. EA, YK participated in the development of the protocol and has primary responsibility for laboratory experiments and contributed to the writing of the manuscript. MD supervised the design and execution of the study, performed the final data analyses. All authors read and approved the final manuscript.
